# Agreement of reported vascular access on the medical evidence report and on medicare claims at hemodialysis initiation

**DOI:** 10.1186/1471-2369-15-30

**Published:** 2014-02-08

**Authors:** Craig A Solid, Allan J Collins, James P Ebben, Shu-Cheng Chen, Arman Faravardeh, Robert N Foley, Areef Ishani

**Affiliations:** 1 United States Renal Data System, Minneapolis Medical Research Foundation, 914 South 8th Street, Suite S4.100, Minneapolis, Minnesota 55404, USA; 2 Department of Medicine, University of Minnesota, Minneapolis, Minnesota USA; 3 Division of Renal Diseases and Hypertension, University of Minnesota, Minneapolis, Minnesota USA; 4 Section of Nephrology, Minneapolis VA Health Care System, Minneapolis, Minnesota USA

**Keywords:** Hemodialysis, Medicare, Validation, Vascular access

## Abstract

**Background:**

The choice of vascular access type is an important aspect of care for incident hemodialysis patients. However, data from the Centers for Medicare & Medicaid Services (CMS) Medical Evidence Report (form CMS-2728) identifying the first access for incident patients have not previously been validated. Medicare began requiring that vascular access type be reported on claims in July 2010. We aimed to determine the agreement between the reported vascular access at initiation from form CMS-2728 and from Medicare claims.

**Methods:**

This retrospective study used a cohort of 9777 patients who initiated dialysis in the latter half of 2010 and were eligible for Medicare at the start of renal replacement therapy to compare the vascular access type reported on form CMS-2728 with the type reported on Medicare outpatient dialysis claims for the same patients. For each patient, the reported access from each data source was compiled; the percent agreement represented the percent of patients for whom the access was the same. Multivariate logistic analysis was performed to identify characteristics associated with the agreement of reported access.

**Results:**

The two data sources agreed for 94% of patients, with a Kappa statistic of 0.83, indicating an excellent level of agreement. Further, we found no evidence to suggest that agreement was associated with the patient characteristics of age, sex, race, or primary cause of renal failure.

**Conclusion:**

These results suggest that vascular access data as reported on form CMS-2728 are valid and reliable for use in research studies.

## Background

In hemodialysis patients, the choice of vascular access type is of primary importance. It is generally accepted that long-term use of catheters can cause vein stenosis [1,2], and catheters are associated with adverse outcomes such as infections and complications [3], compared with arteriovenous fistulas (AVFs) and arteriovenous grafts (AVGs). Additionally, rates of morbidity and mortality are lower for patients who use an internal access (AVF or AVG) [4-15], use of intravenous iron and/or erythropoietin is often lower [16-18], and health care-related costs may be lower [19-22]. Clinical guidelines established by the National Kidney Foundation cite these reasons in advocating use of AVFs whenever possible [23]. The Fistula First campaign was developed to promote use of AVFs for dialysis patients [24,25]. Placement rates for each type of access have changed dramatically over the last 10 years [26], with a decrease in catheter placement rates and an increase in AVF placement rates.

In 2005, the Centers for Medicare & Medicaid Services (CMS) revised the end-stage renal disease (ESRD) Medical Evidence Report (form CMS-2728) to collect information regarding the type of access used at the first outpatient dialysis session for incident patients. While some studies have used the vascular access data from form CMS-2728, lack of validation of those data is cited as a limitation by several authors [27-30]. Previous studies have attempted to validate other data from form CMS-2728, resulting in varying levels of validity and reliability [31-33].

Beginning in July 2010, outpatient dialysis facilities were required to report the type of vascular access used for dialysis on claims submitted to Medicare for payment. This represented the first time that vascular access was reported on Medicare claims, and provided an opportunity to compare the reported access on form CMS-2728 with the reported access on Medicare claims. The purpose of this study was to determine the agreement between the reported vascular access at initiation from these two data sources.

## Methods

### Patients

To ensure the presence of Medicare outpatient dialysis claims at hemodialysis initiation, only patients who were eligible for Medicare before ESRD onset were considered (for patients not Medicare eligible at initiation, Medicare eligibility does not begin until the fourth month of dialysis). Using the United States Renal Data System (USRDS) database, we identified patients aged 67 years or older at the time of initiation who began hemodialysis as their first mode of renal replacement therapy in July through December 2010, and who were Medicare eligible with Medicare as their primary payer. Patients with missing or invalid data from form CMS-2728 were excluded. Eligible patients were also required to have at least one valid Medicare outpatient dialysis claim within 14 days after their ESRD incidence date. Patient characteristics were obtained from the USRDS enrollment database.

### Vascular access

The type of vascular access reported on form CMS-2728 was obtained from question 18d, “What access was used on first outpatient dialysis?” Dialysis claims were identified through the use of revenue center codes (0820 through 0829). To determine the vascular access reported on the first Medicare outpatient dialysis claim after hemodialysis initiation, Healthcare Common Procedure Coding System (HCPCS) modifier codes were used; the modifiers V5, V6, and V7 indicate catheter, AVG, and AVF, respectively.

### Analysis

For each patient, the reported access from each data source was compiled, and the percent agreement represented the percent of patients for whom the access was the same (i.e., catheter, AVF, or AVG). The Kappa statistic was calculated as another measure of agreement [34,35]. Multivariate logistic analysis was performed to identify characteristics associated with the agreement of reported access. The dichotomous response variable was whether or not the reported access from the two data sources agreed (1) or disagreed (0).

Studies conducted under the auspices of the USRDS are exempt from institutional review board approval.

## Results

Of the 13,662 incident hemodialysis patients aged 67 years or older with Medicare coverage at initiation, 211 had an invalid form CMS-2728 or unknown vascular access, and another 3674 lacked an outpatient dialysis claim within 14 days of initiation, resulting in a final study cohort of 9777 patients. The average age was 77.2 years with a range of 67 to 102 years (Table 1). About three-fourths of patients were white, and another 18% were African American. About 8% of patients identified as Hispanic. The most common primary causes of renal failure were diabetes (40%) and hypertension (39%).

**Table 1:** **Patient characteristics (*n *** **= 9777)**

**Variable**	***n *** **or mean (SD)**	**Percent**
Age, years		
Mean	77.2 (6.6)	
67-74	3770	38.6
75-84	4478	45.8
≥ 85	1529	15.6
Sex		
Male	5213	53.3
Female	4563	46.7
Race		
White	7457	76.3
African American	1784	18.2
Asian/Pacific Islander	442	4.5
Other	94	1.0
Ethnicity		
Hispanic	760	7.8
Non-Hispanic	9017	92.2
Primary cause of ESRD		
Diabetes	3910	40.0
Hypertension	3772	38.6
Other/unknown	2095	21.4

*ESRD*, End-stage renal disease; *SD*, Standard deviation.

The distribution of vascular access type identified by each data source and the number of instances of agreement and disagreement appear in Table 2. One patient for whom modifier codes indicated both a catheter and an AVG on the first outpatient dialysis claim was included in the catheter category. The two data sources produced very similar distributions of vascular access use (78% catheter, 18% AVF, 4% AVG), and the two sources agreed on the reported access type for 94% of patients, with a Kappa statistic of 0.83 (*P* = 0.0066, 95% confidence interval 0.82-0.85).

**Table 2:** Agreement of reported access

		**Access reported on first outpatient dialysis claim**			
		**Catheter**	**AVF**	**AVG**	**Total**
Access reported on form CMS-2728	Catheter	7406	189	63	7658 (78.3)
	AVF	200	1490	58	1748 (17.9)
	AVG	34	29	308	371 (3.8)
	Total	7640 (78.1)	1708 (17.5)	429 (4.4)	9777

*Note:* Values are *n* or *n* (%).

*AVF*, Arteriovenous fistula; *AVG*, Arteriovenous graft; *CMS*, Centers for Medicare & Medicaid Services.

Results of the multivariate logistic regression (Table 3) suggest that agreement was not associated with the patient characteristics of age, sex, race, Hispanic ethnicity, or primary cause of renal failure. In fact, the *P* value for the global test for the model was non-significant (likelihood ratio *P* = 0.15), indicating no evidence that any included variable was associated with the agreement in reported vascular access.

**Table 3:** Multivariate logistic regression on the agreement of vascular access

**Variable**	**Estimated odds ratio**	***P***
Male sex	Reference	
Female sex	0.97	0.7296
Age 67–74 years	Reference	
Age 75–84 years	0.92	0.3720
Age ≥ 85 years	1.09	0.5321
White race	Reference	
African American race	0.81	0.0520
Other race	0.80	0.2183
Non-Hispanic ethnicity	Reference	
Hispanic ethnicity	1.26	0.2067

## Discussion

This study represents the first time that vascular access data from form CMS-2728 have been compared with the access type reported on Medicare outpatient claims for the same patients. In general, we found excellent agreement between the two data sources; 94% of the time, the reported access was the same, and the Kappa value of 0.83 is above the threshold of 0.75 typically associated with an indication of excellent agreement [36]. Additionally, from multivariate logistic regression, instances of disagreement appeared to be independent of the patient characteristics of age, sex, race, and primary cause of renal failure. This is encouraging, because it suggests that the validity is likely to be equally strong across different patient populations. Disagreement was more common when an AVF or AVG was reported than when a catheter was reported. However, the use and reporting of catheters far out-number the use and reporting of AVFs or of AVGs, and the distribution of access type reported is in line with the overall distribution. For example, for the 258 cases in which outpatient claims did not agree with AVF access reported on form CMS-2728, 78% of the time the outpatient claims indicated catheter use, which is exactly the percentage of catheters reported overall, and what would be expected if disagreement were random.

The limitations of this study should be noted. The study population was limited to patients aged 67 years or older, and the distribution of the access type used (and reported) may not be representative of the entire incident hemodialysis population. However, the analysis of this population suggested that the validity was not associated with age, and while the results are not generalizable to the population aged younger than 67 years, there is nothing to indicate that validity would differ significantly in a younger population. Another limitation is that form CMS-2728 may be filled out weeks or months after dialysis initiation, with unknown consequences for the reliability of the information on the form. However, for patients in this study, the date of the physician’s signature on the form occurred, on average, 20.4 days after the date that regular dialysis began, and did not differ significantly (*P* = 0.36) for patients with access type agreement (20.5 days) vs. those with lack of agreement (19.6 days) on the first outpatient dialysis Medicare claim. Also, dialysis providers are required to report only the vascular access used for the last hemodialysis session of the month, and reporting the access at each session is at the provider’s discretion. Therefore, in some cases, the access reported on the dialysis claim may not reflect the access used for the first outpatient dialysis session. (In fact, agreement when form CMS-2728 indicates a catheter is slightly worse when the form also indicates presence of a maturing AVF or AVG, possibly reflecting instances of a catheter being used at the first session and an AVF or AVG having matured by the end of the month and thus appearing on the outpatient claim). However, this situation would only serve to increase the difficulty of concordance with form CMS-2728, making our findings all the more encouraging. For researchers hoping to resolve cases in which the data sources disagree, searching for claims involving access procedures (insertions, removals, complications) may provide additional information regarding the type or types of access present at initiation. Finally, this study simply investigated concordance between two data sources, not concordance with type of vascular access actually present, which is ultimately unknown. Future study is warranted, perhaps involving primary data collection regarding the true vascular access used at each dialysis session, to compare with access reported on claims and on form CMS-2728.

## Conclusions

The degree of agreement between form CMS-2728 and outpatient dialysis claims reporting of the type of vascular access used at hemodialysis initiation should give researchers confidence in using data from either source in future studies.

## Abbreviations

AVF: Arteriovenous fistula; AVG: Arteriovenous graft; CMS: Centers for Medicare & Medicaid Services; ESRD: End-stage renal disease; USRDS: United States Renal Data System.

## Competing interests

This study was performed as a deliverable under Contract No. HHSN267200715002C (National Institute of Diabetes and Digestive and Kidney Diseases, National Institutes of Health, Bethesda, Maryland). The authors report no competing interest with this study’s subject matter.

## Authors’ contributions

CS, AC, JE, SC, AF, RF, and AI made substantial contributions to conception and design of the study. JE and SC contributed to acquisition of data. CS, JE, SC, RF, and AI analyzed and interpreted the data. CS, AC, JE, SC, AF, RF, and AI drafted the article or revised it critically for important intellectual content. CS, AC, JE, SC, AF, RF, and AI gave final approval of the version to be published. All authors read and approved the final manuscript.

## Pre-publication history

The pre-publication history for this paper can be accessed here:

http://www.biomedcentral.com/1471-2369/15/30/prepub
